# Risk factors for unintentional injury hospitalisation among Aboriginal and non-Aboriginal children in Australia’s Northern Territory: A data linkage study

**DOI:** 10.1371/journal.pone.0311586

**Published:** 2024-11-05

**Authors:** Jiunn-Yih Su, Vincent Yaofeng He, Anna Lithgow, Steven Guthridge

**Affiliations:** 1 Menzies School of Health Research, Charles Darwin University, Darwin, Australia; 2 Department of Paediatrics, Royal Darwin Hospital, Darwin, Australia; University of Adelaide School of Medical Sciences: The University of Adelaide Adelaide Medical School, AUSTRALIA

## Abstract

**Background:**

Unintentional injuries are a leading cause of hospitalisation for children. This study investigated the incidence and associated risk factors for unintentional injury hospitalisation (UIH) among Aboriginal and non-Aboriginal children aged under 5 years in Australia’s Northern Territory.

**Methods:**

This was a retrospective cohort study using linked data from a perinatal register, hospital admissions, school enrolment and child protection services. The outcome variable was a first UIH. Potential risk factors included gender, pregnancy and birth outcomes, maternal education level, child protection service contact and geographic remoteness. Modified Poisson regression was used for multivariate modelling.

**Results:**

A cohort of 21,189 children (54.0% Aboriginal) born between 2000 and 2010 were followed to the age of 5 years. The overall incidence of first UIH was 25.8 per 1,000 person-years, which was 28.6% higher among Aboriginal than non-Aboriginal children (28.8 and 22.4 per 1000 person-years, respectively). Risk factors identified in the full model included: being male (incidence rate ratio (IRR) 1.26, 95%CI: 1.17–1.36); living in a remote (IRR 1.26, 95%CI: 1.14–1.40) or very remote area (IRR 1.44, 95%CI: 1.29–1.59); having a notification or substantiated notification for abuse (IRR 1.42, 95%CI: 1.27–1.58 and IRR 1.60, 95%CI: 1.41–1.82, respectively); or neglect (IRR 1.32, 95%CI: 1.17–1.48 and IRR 1.28, 95%CI: 1.11–1.47, respectively). After adjustment, there was no difference in UIH rates between Aboriginal and non-Aboriginal children. In both stratified models, being male, living in remote or very remote areas and having a notification or substantiated notification for child maltreatment were identified as risk factors.

**Conclusions:**

Our study found high UIH incidence rates and evidence for an association between UIH and child maltreatment. This suggests child maltreatment and UIH have shared determinants and points to the need for clinicians to be aware of the overlap between these conditions and the importance of cross-agency collaboration in prevention and management.

## Introduction

Unintentional injuries are a leading cause of mortality, morbidity and hospitalisation for children in Australia [1, 2]. Injuries are more likely to be serious and associated with adverse outcomes in children aged under 5 years than in their school aged peers [3]. In 2017–2018, a total of 19,629 children, aged 1–4 years, were hospitalised across Australia due to unintentional injuries [4]. The hospitalisation rate was 1,545 cases per 100,000 population and was higher for boys than girls (1,758 versus 1,321 per 100,000 population). The top three causes for unintentional injury hospitalisation (UIH) in this age group were falls, exposure to inanimate mechanical forces and accidental poisoning [4]. The occurrence and associated morbidity and mortality of unintentional injuries are not evenly distributed among subgroups of the populations, including higher rates reported for Aboriginal and Torres Strait Islander (henceforth respectfully referred to as Aboriginal) children than their non-Aboriginal counterparts [1, 2, 5–7]. Similar inequalities have also been reported for First Nations populations in other countries [8–13]. Moreover mortality rates from various types of unintentional injuries have been reported to be up to five times higher among Aboriginal children than non-Aboriginal children [14]. A recent data linkage study conducted in Australia’s New South Wales (NSW) reported Aboriginal children aged 0 to 13 years were 1.6 times more likely than non-Aboriginal children to be hospitalised for unintentional injuries (157.33 and 99.30 per 10,000 person-years, respectively) [5]. In the Northern Territory (NT) of Australia, during the period of 2001–2011, the age-standardised hospitalisation rate for falls (the most common type of unintentional injuries) for Aboriginal children was 1.13 times the rate for non-Aboriginal children (age standardised rates 722.9 and 637.2 per 100,000 population, respectively) [15]. In addition, the rates for all types of unintentional injuries have been reported to be higher in the Central Australia region of the NT than the northern or “Top End” region for both Aboriginal and non-Aboriginal children [15].

Prior to 1940, there was a general acceptance that injuries were caused by ‘carelessness, stupidity or indifference’, and the term *accidental* was used to refer to injuries that were not intentional [16]. As the understanding of the aetiology and epidemiology of injury evolved researchers began describing injuries, like infectious diseases, as an outcome of interactions between host (the person sustaining the injury), environment (external factors) and injury agents (the forces that cause the bodily harm); and the term *unintentional* replaced *accidental* when referring to such injuries [17].

Moreover, research has shown that some attributes and behaviours of both the child and their parents or caregivers can impact on children’s risk of unintentional injury. Child factors (the host) found to increase injury risk include being male, younger age, impulsive and hyperactive behaviour, while risk factors concerning parents include younger age, single parent, unemployment, lower education levels, and inadequate supervision [18–23]. Parental supervision or parenting styles and children’s behaviour can moderate each other in their associations with injury risk [23, 24]. In addition, factors concerning children’s living environment can increase children’s injury risk, including socioeconomic disadvantage, neighbourhood (examples are high traffic volume, availability of safe play areas and unsafe roads) and home environments (examples are crowdedness and safety structures such as staircase rails, exposure to domestic violence and unsafe living environments) [16, 18, 25, 26]. There are also other factors that are specific to Aboriginal children, such as ongoing social impacts of colonisation, past social policies including forced removal of children and living in isolated areas [5, 14].

The impact of some of the factors listed above may be mediated by child maltreatment. For instance, a study by Mooney [27] found that socioeconomic status is correlated with instances of child maltreatment, both of which are found to be associated with higher injury risk. Among the four types of child maltreatment (neglect, sexual abuse, physical abuse and emotional abuse), [28] neglect commonly involves a lack of parental supervision while emotional abuse may impact on emotional development in children, with both types of child maltreatment likely to facilitate an increase in children’s risk of unintentional injury [23, 29, 30]. Physical and sexual abuse are less likely to be associated with UIH in children, however, the injuries they cause can sometimes be misclassified as unintentional [29, 31, 32]. These findings are particularly important in the investigation of risk factors for unintentional injury in Aboriginal children in the NT, where there are substantially higher rates of child protection notifications and substantiations compared with other states and territories of Australia. For instance, a recent report revealed that, in 2018–19, the rate of substantiated notification was 18.8 per 1000 population in the NT, compared with 8.5 per 1000 for Australia as a whole; the population rate of substantiated notification of child maltreatment was 9 times higher in Aboriginal children than in their non-Aboriginal counterparts; and neglect and emotional abuse are by far the most common types of child maltreatment notification [33].

Most unintentional injuries are preventable. Preventing unintentional injuries in Aboriginal children will contribute towards reducing the associated mortality and, in turn, the first target of the new National Agreement on Closing the Gap, signed in 2020 by all Australian governments and the Coalition of Aboriginal and Torres Strait Islander peak organisations, namely, closing the gap in life expectancy within a generation, by 2031 [34]. However, the formulation of strategies for injury prevention is best informed by evidence from research that investigates the risk factors associated with unintentional injury in the targeted population. Many published Australian studies: have either focused on a single type of unintentional injury (such as transport, [35] drowning, [36] burns [37–39] and poisoning [40]); have been primarily designed for investigating incidence rates (thus not for risk factors), [15, 38, 39, 41] or the gap between Aboriginal and non-Aboriginal children; [5–7, 12] covered a wide age range (such as 0–13 years), [5, 6] or did not include stratified analysis for sub-populations including by Aboriginal status. Evidence from these studies, while useful, may not be suitable for informing policies and interventions specific for injury prevention in different groups of children such as those aged under 5 years. To date, there have been few studies that have used linked population-level data to investigate the risk factors for unintentional injury hospitalisation (UIH) in children, [12] and no studies have been conducted in the NT.

This study was conducted to fill this gap in knowledge. The aims of the study were: a) to estimate the incidence of UIH in NT Aboriginal and non-Aboriginal children aged under 5 years; and b) to investigate the risk factors for UIH in the study population.

## Materials and methods

### Study design and setting

This study was a retrospective cohort study using linked administrative datasets to investigate the occurrences of and risk factors for UIH in children aged under 5 years. Covering an area of 1.3 million square kilometres in the northern and central part of Australia, the NT is sparsely populated with a population of 245,678, which is the smallest among all Australian states and territories [42]. The NT also has the highest proportion of Aboriginal people (29.5%, compared with 3.3% for Australia) [42] and the highest proportion of people living in remote and very remote areas (40.4% in 2017) when compared to other jurisdictions [43]. The majority of the Aboriginal population live in remote communities, where health care is usually delivered by a community clinic staffed by remote area nurses, midwives and Aboriginal health practitioners and supported by medical officers, specialists and allied health professionals visiting from larger centres.

### Study cohort and data sources

The study cohort was defined as children born in the NT from 1 January 2000 to 31 December 2010, for whom there was evidence of NT residence up to the age of 5 years. Individual-level linked records from five administrative datasets were retrieved in January 2021, from a comprehensive data repository containing a total of fourteen administrative datasets [44]. All data were de-identified. Authors had no access to information that could identify individual participants during or after data collection. The linkage was undertaken by SA NT DataLink using a combination of probabilistic linkage and clerical review of uncertain matches [45, 46]. A 99.6% accuracy was reported in a recent empirical linkage quality assessment review for the SA NT Datalink [47].

The *NT Perinatal Data Register* dataset was used to select NT born children. This dataset is a statutory collection of maternal and perinatal information for all births in the NT and was available from 1994 to 2014. The quality and completeness of data within the NT Perinatal Data Register is under continuing review, including by comparison with the NT Births Register and by quality testing through collation within the national Perinatal Data Collection. Information on deaths in the NT was retrieved from the *NT Death Register*, a statutory administrative data collection managed by the NT Government, which contained records of all deaths reported in the NT. We used children’s Year 1 school attendance records, when aged about 6 years, from the *NT Government school dataset* as evidence for their presence in the NT at age 5. This dataset contains enrolment and daily attendance records of students attending NT public schools and was available from 2005 to 2016. Utilisation of this dataset ensured a high level of accuracy and comprehensive follow-up of the study population of the NT, where interstate migration of non-Aboriginal children is common [48] and school enrolment at age 6 is compulsory [49]. Information on enrolment in non-government schools was not available.

Data for UIHs were retrieved from the *NT Hospital Separations dataset*. This dataset contains diagnosis and procedure information for all hospital admissions in all six NT public hospitals and was available for the period of 2000–2017 (it is uncommon for children to be admitted to the one private hospital in the NT).

Child maltreatment data were retrieved from the *NT Child Protection* dataset, which contains statutory records of children with contacts with government child protection services during the period of 1998–2018. This dataset includes information on all notifications of possible maltreatment, substantiated cases and placements in out-of-home care. In the NT, reports of suspected child maltreatment made to child protections services are recorded as a ‘notification’. After assessment, notifications may be referred for investigation resulting in some notified cases being substantiated as child abuse or neglect, which are recorded as ‘substantiations’ in the database.

Non-residents, stillbirths and children whose gender was recorded as ‘indeterminant’ or died before the age of 5 years without experiencing UIH were excluded. Children without enrolment or attendance records in NT Government schools for Year 1 were deemed to have migrated out of the NT or to be attending non-government schools.

### Outcome measure

The outcome measure was ‘the first UIH before the age of 5’. This was chosen to facilitate the investigation of risk factors for children ever experiencing UIH. Unintentional injuries are also treated in other health care settings, including in NT remote and urban clinics, however there was no population-wide data source to extend the analysis to these settings. A UIH was defined as a record of hospitalisation (data retrieved the *NT Hospital Separations dataset*) with its principal diagnosis code falling in the range ‘S00-T75’ or T79 plus an external cause code from the range V01-X59 or Y85-Y86 in any other diagnosis field [5, 9]. The former is the injury diagnosis code describing the physical injury and its related complications while the latter describes the mechanism, circumstances and other characteristics of the incident that caused them. We conducted analyses on overall UIHs and also on selected major groups of external causes (adapted from the injury matrix produced by the Center for Disease Control and Prevention of the United States (S1 Appendix) [50].

### Covariates

Relevant covariates were selected from the study datasets. As there was inconsistency between datasets for the recorded Aboriginal status for some children, we used a derived variable on Aboriginal status created using a hierarchical algorithm, described elsewhere, [51] and based on the demonstrated accuracy of Aboriginal status with data in the health datasets being the most accurate [52].

Maternal, antenatal and perinatal factors retrieved from the *NT Perinatal Data Register* dataset included gender, teenage pregnancy (maternal age<20 at birth), maternal diabetes and hypertension, maternal smoking and alcohol consumption during pregnancy, less than 7 antenatal visits, low birth weight (<2500 grams), preterm birth (<37 weeks gestational age) and twin birth. Parental factors retrieved from the *NT Government school* dataset included mother’s education level.

For the child maltreatment factors, we used data from *NT Child Protection* dataset to construct two categorical variables, ‘neglect’ for notifications and substantiations with the primary type being neglect and ‘abuse’ for those with the primary type being emotional, sexual or physical abuse. Each variable consisted of three categories: none (no notifications or substantiations), notified only (having notifications but no substantiations), substantiated (having one or more substantiated notifications). We included all records before the age of 5 years in constructing these two variables without considering the temporal order in relation to the first UIH. This was because we construed them as child maltreatment factors that may impact on the occurrence of UIH as well as on factors representing the background condition of parental care during the early childhood period.

Two variables concerning the living environment were included in the analysis. The level of relative remoteness as measured with the Accessibility and Remoteness Index of Australia (ARIA+; there were only ‘outer regional’, ‘remote’ and ‘very remote’ categories in the study cohort) [53]. Considering the higher reported rates of UIH in Central Australia compared with Top End and mentioned above, [15] we included a binary variable of living in Central Australia (vs the reference category of Top End) based on mother’s place of residence at time of birth and recorded in perinatal data. We were unable to include socioeconomic status data as a covariate because the inconsistent and incomplete recording of resident location in the *NT Perinatal Data Register* dataset prevented mapping area-level socioeconomic status using the ‘Socio-economic Indexes for Areas’ from the Australian Bureau of Statistics.

### Statistical analysis

Demographic data, the included covariates and proportions of children with UIH were examined for the whole study cohort and compared between Aboriginal and non-Aboriginal children. The distribution of UIH by external cause groups in these two subgroups was also compared.

The follow-up period for each individual child started from their birth and ended at the earliest of the following time points: the first UIH, the child’s fifth birthday or the end date of follow-up (31 December 2017). The length of follow-up for children who had Year 1 attendance records but no UIH was set as 5 person-years. Incidence rates were calculated by dividing the number of children with UIH by the sum of follow-up time for the study cohort, Aboriginal children and non-Aboriginal children, respectively.

Univariate analyses were performed with Chi-square test or Poisson regression as appropriate. Multivariate analyses were performed using modified Poisson regression, which used robust variance estimation to avoid overestimating standard errors of the estimated relative risk [54]. We built multivariate regression models by first including all covariates with unadjusted p< 0.25 and then removing covariates with an adjusted p ≥ 0.05 from the model until a stable and parsimonious model was achieved [55, 56]. Interaction terms between gender, remoteness and categories of abuse and neglect were examined during the model building process. Gender and Aboriginal status were retained as control variables throughout the model building process. We checked for multicollinearity of the independent variables and the outcome variables using Variance Inflation Factor (VIF). All analyses were conducted using Stata version 15 (Stata Corporation, College Station, TX, USA). A two-tailed p value <0.05 or an incidence rate ratio (IRR) with 95% confidence interval (CI) not inclusive of the unity was considered significant.

### Ethics approval and consent to participate

Ethics approval to conduct this research was obtained from the Human Research Ethics Committee of the Northern Territory Department of Health and Menzies School of Health Research (HREC-2016-2708). A waiver of consent was approved for this study which is based on de-identified population level data. The concept and written research plan for this research was reviewed, and the final version of the manuscript was presented to and supported by the First Nations Advisory Group for the Centre for Child Development and Education. The advisory group includes community members.

## Results

### Descriptive statistics

**Fig 1:**
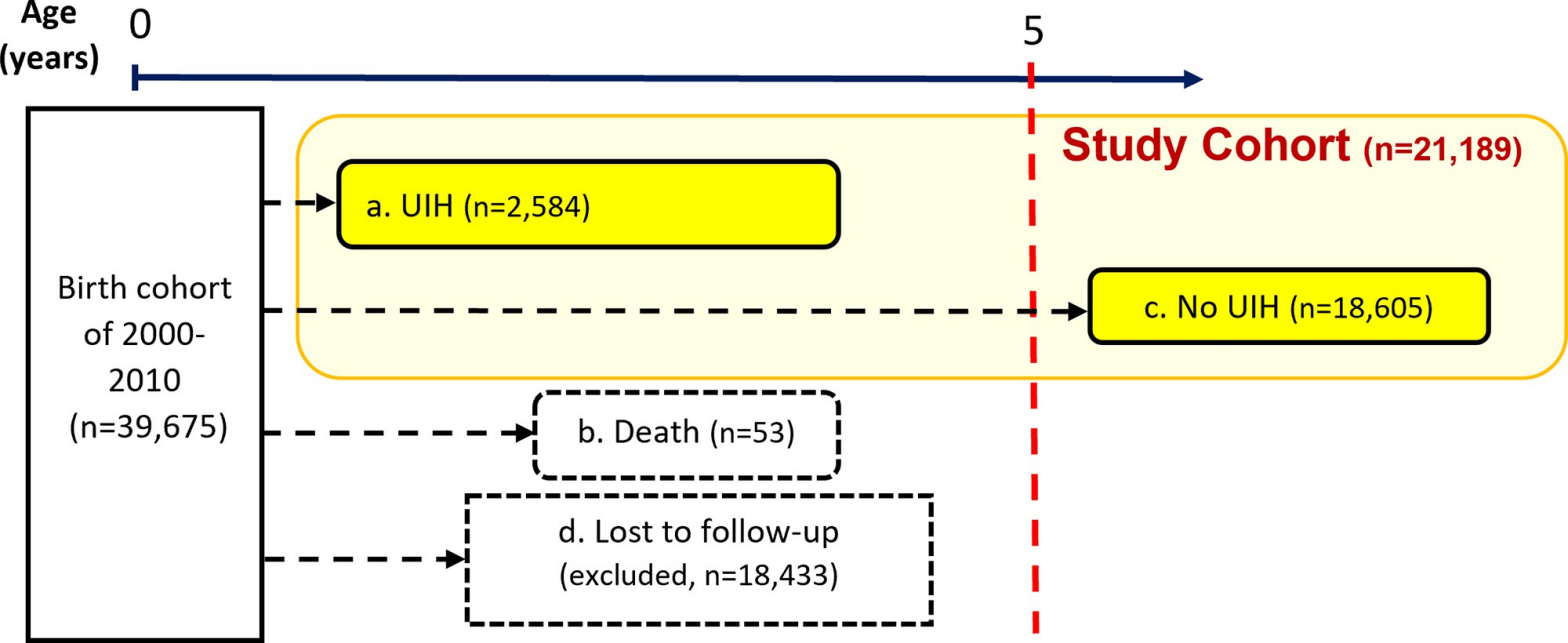
The process of study cohort selection.

A total 39,675 children born in the NT between 2000 and 2010 were identified in the Perinatal Data Register (Fig 1). After excluding 53 children who died before the age of 5 and 18,433 children for whom there was no evidence of enrolment in an NT government school, the remaining 21,189 children were selected into the study cohort. The study cohort included 11,442 (54.0%) Aboriginal children. Among the 21,189 children in the study cohort, there was evidence of difference between Aboriginal and non-Aboriginal children for all factors examined (Table 1). Aboriginal children had a higher proportion of: being male; living in remote or very remote areas; living in Central Australia; having a maternal or perinatal risk factor; and having notifications and substantiated notifications for abuse and neglect.

**Table 1:** Descriptive statistics of Aboriginal and non-Aboriginal children in the study cohort.

Variable		Aboriginal (n = 11442)		Non-Aboriginal (n = 9747)		*p* value
		n =	%	n =	%	
**Demography**						
	***Male***	5959	52.1	4939	50.7	0.041
	***Remoteness***					<0.0005
	Outer regional	2453	21.4	7286	74.8	
	Remote	3394	30.0	1863	19.1	
	Very remote	5595	48.9	598	6.1	
	***Top End/Central Australia split***					<0.0005
	Top End	8052	70.4	8451	86.7	
	Central Australia	3390	29.6	1296	13.3	
**Maternal**						
	***Maternal age<20***	3040	26.6	475	4.9	<0.0005
	***Antenatal visits<7***	4006	35.8	1466	15.2	<0.0005
	***Diabetes***	1044	9.1	543	5.6	<0.0005
	***Hypertension***	536	4.7	226	2.3	<0.0005
	***Drank alcohol during pregnancy***	1221	12.9	729	8.1	<0.0005
	***Smoked during pregnancy***	4711	49.7	1917	21.4	<0.0005
	***Parity***					<0.0005
	<2	6494	56.8	7180	73.7	
	2–3	3481	30.4	2219	22.8	
	4+	1463	12.8	343	3.5	
**Perinatal**						
	***Twin birth***	215	1.9	269	2.8	<0.0005
	***Pre-term birth***	1507	13.2	682	7.0	<0.0005
	***Low birthweight (<2500 grams)***	1410	12.3	581	6.0	<0.0005
	***Caesarean birth***	1959	17.1	1383	14.2	<0.0005
		5959		4939		
**Child maltreatment**						
	***Abuse***					<0.0005
	No	8543	74.7	8989	92.2	
	Notified only	1784	15.6	589	6.0	
	Substantiated	1115	9.7	169	1.7	
	***Neglect***					<0.0005
	No	8908	77.9	9319	95.6	
	Notified only	1480	12.9	342	3.5	
	Substantiated	1054	9.2	86	0.9	
**Unintentional injury hospitalisation**						
	***Having any UIH***	1553	13.6	1031	10.6	<0.0005
	***Age at first UIH (years)***					<0.0005
	<2	460	29.6	441	42.8	
	2–3	715	46.0	420	40.7	
	4+	378	24.3	170	16.5	
	***Median age at first UIH***	*** ***	2.9 years		2.3 years	

To assess for selection bias associated with the use of NT Government school Year 1 enrolment and attendance records for cohort selection, we compared demographic and perinatal variables of children selected into the study cohort with those who were excluded. The major difference between those selected and those excluded was the proportion of Aboriginal and non-Aboriginal children, with the relatively similar proportions in the study cohort (54.0% Aboriginal children, 46.0% non-Aboriginal children) compared with the excluded population (26.2% Aboriginal children, 73.8% non-Aboriginal children). There were fewer substantial differences in characteristics recorded at birth in a separate comparison of Aboriginal and non-Aboriginal children, in and out of the study cohort (S2 Appendix). For both groups there were differences in relative remoteness of the residence of mother at time of birth, maternal age and proportion of mothers with fewer than seven antenatal care visits. For Aboriginal children in the study cohort there was a lower proportion of pre-term birth (13.2% v 16.1%) and low birthweight (12.3% v 14.8%) compared to those excluded from the study. For non-Aboriginal children, differences in characteristics for those in the study cohort compared to those excluded included: higher proportions recording alcohol consumption (7.5% v 6.0%), smoking in pregnancy (19.7% v 14.2%) and lower parity of mother at time of birth.

The study cohort consisted of 2,584 children with one or more UIHs and 18,605 with no record of a UIH, before the age of 5 or the end date of follow-up. The proportion of children with UIH was higher among Aboriginal children (13.6% (95% CI: 13.0–14.2%) vs 10.6% (95%CI: 10.0–11.2%), p<0.0005). For the study cohort, the incidence rate of UIH was 25.8 per 1,000 person-years. The incidence rate for Aboriginal children was 28.6% higher than the non-Aboriginal rate (28.8 and 22.4 per 1,000 person-years, respectively). The distribution by the age at first UIH was also different between the two populations with the first UIH occurring at older ages for Aboriginal children (median age at first UIH 2.9 years vs 2.3 years).

As shown in Table 2, the external cause group accounting for the highest proportion of records of the first UIH was falls in both populations, followed by contact with fire/heat. There was evidence for a difference in UIH rates between Aboriginal and non-Aboriginal children in the distribution of children across the external cause groups (Chi-square test, p<0.0005). Incidence rates were higher among Aboriginal children for transport, struck by or against, cut/pierce, natural or environmental and contact with fire/heat. Rates among non-Aboriginal children were higher than Aboriginal children for drowning only. There was no evidence for a difference in rates between Aboriginal and non-Aboriginal children for falls, poisoning and suffocation.

**Table 2:** The proportion of children who experienced unintentional injury hospitalisation by external causes and Aboriginal status, Northern Territory.

Cause of UIH	Aboriginal (n = 11442)			Non-Aboriginal (n = 9747)					
	n =	%	95% CI	n =	%	95% CI	IRR	95% CI	p value
**All UIHs**	**1553**	13.6%	(12.9–14.2%)	**1031**	10.6%	(10.0–11.2%)	1.28	(1.19–1.38)	<0.0005
Transport	94	0.8%	(0.7–1.0%)	50	0.5%	(0.4–0.7%)	1.60	(1.14–2.25)	0.007
Falls	411	3.6%	(3.3–4.0%)	380	3.9%	(3.5–4.3%)	0.92	(0.80–1.06)	0.241
Struck by or against	121	1.1%	(0.9–1.3%)	65	0.7%	(0.5–0.8%)	1.59	(1.17–2.14)	0.003
Cut/pierce	88	0.8%	(0.6–0.9%)	34	0.3%	(0.2–0.5%)	2.20	(1.49–3.27)	<0.0005
Poisoning	118	1.0%	(0.9–1.2%)	93	1.0%	(0.8–1.2%)	1.08	(0.82–1.42)	0.573
Natural or environmental	109	1.0%	(0.8–1.1%)	61	0.6%	(0.5–0.8%)	1.52	(1.11–2.08)	0.008
Drowning	22	0.2%	(0.1–0.3%)	34	0.3%	(0.2–0.5%)	0.55	(0.32–0.94)	0.029
Suffocation	15	0.1%	(0.1–02%)	22	0.2%	(0.1–0.3%)	0.58	(0.30–1.12)	0.104
Contact with fire/heat	297	2.6%	(2.3–2.9%)	99	1.0%	(0.8–1.2%)	2.56	(2.04–3.20)	<0.0005
Others	278	2.4%	(2.2–2.7%)	193	2.0%	(1.7–2.3%)	1.23	(1.02–1.47)	0.027

Notes: UIH: unintentional injury hospitalisation; CI: confidence interval.

### Statistical modelling for risk factors

In univariate analysis (Table 3), the following factors had evidence of an association with higher incidence rates of UIH compared with their respective reference category: being Aboriginal compared with non-Aboriginal, male gender, living in remote or very remote areas (vs outer regional), living in Central Australia (vs Top End), maternal age <20, maternal attendance at less than 7 antenatal visits, maternal alcohol consumption or smoking during pregnancy, maternal gravidity of 4 or more, having notifications or substantiated notification for abuse or neglect.

**Table 3:** Results of univariate and multivariate analyses, combined models.

Variable		n =	% with UIH	Univariate analysis			Multivariate analysis		
				IRR_unadj_	95% CI	P_unadj_	IRR_adj_	95% CI	P_adj_
	*** ***			** **	** **	** **	** **	** **	** **
**Aboriginal status**				** **	** **	** **	** **	** **	** **
	*Non-Aboriginal*	9747	10.6	Ref.			Ref.		
	*Aboriginal*	11442	13.6	1.28	(1.19–1.38)	<0.0005	0.93	(0.85–1.02)	0.148
**Gender**				** **	** **	** **	** **	** **	** **
	*Female*	10291	10.7	Ref.			Ref.		
	*Male*	10898	13.6	1.27	(1.18–1.36)	<0.0005	1.26	(1.17–1.36)	<0.0005
**Remoteness**									
	Outer regional	9739	10.1	Ref.			Ref.		
	Remote	5257	13.1	1.30	(1.18–1.42)	<0.0005	1.26	(1.14–1.40)	<0.0005
	Very remote	6193	14.7	1.46	(1.34–1.58)	<0.0005	1.44	(1.29–1.59)	<0.0005
**Region**									
	Top End	16503	10.9	Ref.					
	Central Australia	4686	16.8	1.55	(1.43–1.67)	<0.0005			
**Maternal**									
	***Maternal age<20***								
	No	17673	11.9	Ref.					
	Yes	3515	13.9	1.18	(1.07–1.29)	<0.0005			
	***Antenatal visits<7***								
	No	15389	11.7	Ref.					
	Yes	5472	13.7	1.17	(1.08–1.26)	<0.0005			
	***Diabetes***								
	No	19602	12.1	Ref.					
	Yes	1587	12.5	1.02	(0.89–1.17)	0.721			
	***Hypertension***								
	No	20427	12.2	Ref.					
	Yes	762	12.5	1.02	(0.84–1.24)	0.815			
	***Alcohol consumption during pregnancy***							
	No	16516	11.9	Ref.					
	Yes	1950	13.5	1.14	(1.01–1.28)	0.037			
	***Smoked during pregnancy***								
	No	11815	11.4	Ref.					
	Yes	6628	13.1	1.15	(1.06–1.24)	0.001			
	***Parity***								
	<2	13674	11.9	Ref.					
	2–3	5700	12.5	1.06	(0.97–1.15)	0.184			
	4+	1806	13.7	1.15	(1.02–1.31)	0.025			
**Perinatal**									
	***Twin birth***								
	No	20705	12.2	Ref.					
	Yes	484	12.4	1.02	(0.80–1.29)	0.891			
	***Pre-term birth***								
	No	18997	12.1	Ref.					
	Yes	2189	13.1	1.08	(0.96–1.21)	0.188			
	***Low birthweight (<2500 grams)***								
	No	19195	12.1	Ref.					
	Yes	1991	12.7	1.05	(0.93–1.18)	0.464			
	***Emergent Caesarean birth***								
	No	17847	12.1	Ref.					
	Yes	3342	12.6	1.04	(0.94–1.15)	0.438			
**Child maltreatment**									
	***Abuse***								
	No	17532	11.0	Ref.			Ref.		
	Notified only	2373	16.9	1.54	(1.39–1.70)	<0.0005	1.42	(1.27–1.58)	<0.0005
	Substantiated	1284	20.4	1.86	(1.66–2.09)	<0.0005	1.60	(1.41–1.82)	<0.0005
	***Neglect***								
	No	18227	11.2	Ref.			Ref.		
	Notified only	1822	17.8	1.58	(1.43–1.77)	<0.0005	1.32	(1.17–1.48)	<0.0005
	Substantiated	1140	18.6	1.66	(1.46–1.88)	<0.0005	1.28	(1.11–1.47)	0.001

Notes: UIH: unintentional injury hospitalisation; IRR: incidence rate ratio; Ref: reference category; adj: adjusted; unadj: unadjusted; CI: confidence interval.

For multivariate modelling, we purposely retained two variables–Aboriginal status and being male, to control for them. After testing other variables only remoteness and child maltreatment factors remained in the final parsimonious model (Table 3). The incidence rate of UIH was higher in boys (IRR 1.26, 95% CI: 1.17–1.36, p<0.0005); those living in either remote (IRR 1.26, 95% CI: 1.14–1.40, p<0.0005) or very remote areas (IRR 1.44, 95% CI: 1.29–1.59, p<0.0005); those with notifications for abuse (IRR 1.42, 95% CI: 1.27–1.58) or substantiated notifications for abuse (IRR 1.60, 95% CI: 1.41–1.82); those who had notifications for neglect (IRR 1.32, 95% CI: 1.17–1.48) or substantiated notifications for neglect (IRR 1.28, 95% CI: 1.11–1.47). Notably, there was no evidence for an independent effect of Aboriginal status (0.93, 95%CI: 0.85–1.02, p = 0.148). The lack of difference based on Aboriginal status and the substantial differences between Aboriginal and non-Aboriginal children for all other factors supported different risk profiles for the two groups requiring a stratified analysis by Aboriginal status. In the stratified multivariate analysis for Aboriginal children the factors associated with higher incidence rates were: being male, living in a very remote area, having notifications or substantiated notifications for abuse or for neglect (Table 4). In contrast, for non-Aboriginal children, higher IRRs were associated with: being male, living in remote areas and having notifications for abuse.

**Table 4:** Results of multivariate analyses, stratified by Aboriginal status.

Variable			Aboriginal (n = 11442)					Non-Aboriginal (n = 9747)			
		n =	% with UIH	IRR_unadj_	(95% CI)	P_unadj_	n =	% with UIH	IRR_unadj_	(95% CI)	P_unadj_
**Gender**			** **	** **	** **	** **	** **	** **	** **	** **	** **
	*Female*	5483	12.1	Ref.			4808	9.1	Ref.		
	*Male*	5959	14.9	1.23	(1.12–1.35)	<0.0005	4939	12.0	1.32	(1.17–1.48)	<0.0005
**Remoteness**											
	Outer regional	2453	12.4	Ref.			7286	9.4	Ref.		
	Remote	3394	11.8	0.97	(0.84–1.12)	0.685	1863	15.4	1.66	(1.46–1.88)	<0.0005
	Very remote	5595	15.2	1.30	(1.15–1.47)	<0.0005	598	10.5	1.14	(0.89–1.46)	0.288
**Child maltreatment**											
	***Abuse***										
	No	8543	11.7	Ref.			8989	10.3	Ref.		
	Notified only	1784	18.0	1.47	(1.30–1.66)	<0.0005	589	13.4	1.32	(1.05–1.66)	0.019
	Substantiated	1115	21.3	1.66	(1.45–1.90)	<0.0005	169	14.8	1.35	(0.91–2.00)	0.136
	***Neglect***										
	No	8908	12.0	Ref.			9319	10.5	Ref.		
	Notified only	1480	19.1	1.38	(1.21–1.56)	<0.0005	342	12.3	1.04	(0.76–1.42)	0.822
	Substantiated	1054	19.0	1.30	1.12–1.50)	<0.0005	86	14.0	1.08	(0.61–1.93)	0.782

Notes: UIH: unintentional injury hospitalisation; IRR: incidence rate ratio; Ref: reference category; adj: adjusted; unadj: unadjusted; CI: confidence interval.

## Discussion

Our study found that, among NT-born children aged under 5 years, Aboriginal children had a higher risk of being hospitalised for unintentional injury than their non-Aboriginal counterparts. However, after controlling for all covariates, retained in the final multivariate model, there was no evidence of a difference in incidence rate between the two groups. This suggests that any difference in rates, between populations, can be explained by other factors included in the statistical model, including being male, living in a remote or very remote area or having a record of either notification or a substantiated notification for child abuse or neglect. Rates for both NT Aboriginal and non-Aboriginal children were considerably higher than the corresponding rates reported for children aged 0–13 years in NSW (15.7 per 1,000 person-years for Aboriginal children and 9.9 for non-Aboriginal ones) [5].

Our results highlight the important role of child maltreatment in the risk profiles for UIH in both Aboriginal and non-Aboriginal children. In Aboriginal children, having a substantiated notification for abuse or neglect increased the risk of UIH (by 60% and 20% respectively). Among non-Aboriginal children, having a substantiated notification for abuse increased the risk of UIH by 40%. Although maltreatment has been identified as an important risk factor for both mortality and morbidity in children due to unintentional injury, [57–59] our study has provided direct evidence for this association, which was previously not available for children in the NT.

This finding has several implications for policy and practice for both injury prevention and child protection. Firstly, it is important for both primary care and hospital clinicians to be aware of the increased risk of child maltreatment (either as a cause or as an associated condition) when treating children with unintentional injuries. Scott et al. have previously highlighted that, in a child protection context, a child presenting with repeated injuries may be a warning that these injuries may have been caused by neglect and warrant further investigation [30]. For some children, contact with clinicians when hospitalised may be the only chance for maltreatment to be recognised. Considering the high rates of child maltreatment reported in the NT, especially among Aboriginal children, it is important for clinicians to take a detailed history of the social and family context and a more detailed understanding of the injury events. This may facilitate the detection of parents/caregivers with patterns of parental behaviours or parenting styles that may increase the risk of injury. Where risks are identified, clinicians should take the initiative to refer the child to child protection and family support services.

Secondly, the increased risk of UIH associated with both neglect and abuse types of child maltreatment points to the importance of providing injury prevention counselling to parents/caregivers of at-risk children in injury prevention interventions. In a review of evidence on unintentional injury, parental supervision and prevention, Robinson et al. reported that for children below the age of five parents are most often the target audience of injury prevention strategies [2]. They found that holistic home visiting programs targeting parents from socio-economically disadvantaged backgrounds and incorporating education around child safety, such as the Nurse-Family Partnerships Program, can be effective in reducing child injuries. A Cochrane review also found that parenting interventions for at-risk families appeared to be effective in reducing self-reported or medically-attended injury in children when implemented as a component of multi-faceted interventions [60]. In the NT context specifically, Robinson et al. stated that although there is overlap between cases of child injury risk and cases of supervisory neglect, child protection responses may not be the appropriate path to respond to child injury risk due to the high thresholds of notification and substantiation of cases of neglect [2]. Therefore, multifaceted community interventions with high levels of community engagement around key issues in child safety and wellbeing are the important foundation upon which surveillance and preventive measures need to be implemented for primary and secondary prevention of child injuries.

Living in remote or very remote areas was another risk factor identified for both Aboriginal and non-Aboriginal children. Aboriginal children, living in very remote areas had a 30% increased risk of UIH and non-Aboriginal children living in remote areas had 70% increased risk. Children living in these areas may have a greater risk of more severe injuries (that required hospitalised treatment); or from injury complications as a result of delayed access to hospitals which are all located in major population centres; or may have less adult supervision or engage in higher risk activities. It may also be because there are fewer health services in remote locations and relatively mild injuries that may need to be referred to hospitals for treatment. In any case, this is a factor that should be considered in formulating policy and practice for injury prevention.

### Strengths and limitations

One of the strengths of our study was its use of multiple linked administrative datasets, which enabled the inclusion of a range of potential risk factors in the analysis. The use of Year 1 school attendance records made it possible to follow the study cohort from birth to age 5, which was important for the NT where there is a high level of interstate migration of non-Aboriginal children. In addition, the inclusion of linked child protection data allowed the investigation of the independent association between child maltreatment and UIH. To our knowledge, our study was the first study in Australia to include child maltreatment in the investigation of risk factors for UIH.

Our study has several limitations. Firstly, the study cohort was based on a birth cohort with a record of continuing residence in the NT demonstrated by NT Government school records. The high level of interstate migration of non-Aboriginal families in the NT, has resulted in a disproportionate loss in the study cohort. There would also be children excluded from the study cohort but who remained resident and attended non-government schools, including Catholic and independent schools. While primary school education is compulsory in the NT, a small proportion of children may not have been enrolled at Year 1. The extent to which these exclusions may have biassed the results is uncertain, however comparison of the demographic and perinatal characteristics, including lower rates of premature birth and low birthweight, suggests that the Aboriginal study cohort, may be at lower risk than the general population of Aboriginal children and therefore the study results may be a slight underestimate of true results. By contrast the similar comparison for non-Aboriginal children suggests the study cohort of non-Aboriginal may be at slightly higher risk. A second limitation is that the outcome measure of ‘the first UIH before the age of 5’ the study used precluded the inclusion of overall incidents of UIH in the calculation of incidence. Further, the study was based on more severe, non-fatal injuries which require hospital admission. The identified risk factors may not be the same for less severe injuries managed in a primary care setting. We plan a follow-up study for risk factors for unintentional injuries utilising primary care data for Aboriginal children living in remote communities.

## Conclusions

This study demonstrated higher rates of UIH in Aboriginal children compared with their non-Aboriginal counterparts, a difference fully explained by the varying exposures to the modelled risk factors. Being male, living in remote or very remote areas and a record of one or more child protection notifications for child abuse were risk factors for UIH for both populations, while a child protection notification for neglect was a risk factor for UIH for Aboriginal children, only. These findings suggest that there are shared determinants of child maltreatment and UIH and point to the importance of cross-agency collaboration in prevention and management. In the context of UIH, clinicians should maintain a high awareness of the overlap between these conditions. Community-based parenting interventions targeting at-risk families and providing education around child safety should be implemented to reduce injury risk.

## Supporting information

S1 Appendix Table 1: ICD-10-AM codes for external cause groups of injury mechanisms. (DOCX)

S2 Appendix Table 2: Comparison of demographic and perinatal characteristics between the study cohort and the excluded cohort, stratified by Aboriginal status. (DOCX)
