# Supplementary material for: Risk factors for unintentional injury hospitalisation among Aboriginal and non-Aboriginal children in Australia’s Northern Territory: A data linkage study
Source: PLoS One. 2024 Nov 5;19(11):e0311586. doi: 10.1371/journal.pone.0311586 (PMC11537399; doi:10.1371/journal.pone.0311586)
Supplement: S1 Appendix — (DOCX) [file pone.0311586.s001.docx]

Appendix Table 1: ICD-10-AM codes for external cause groups of injury mechanisms

| **Injury mechanism** | **ICD-10-AM code** |
| --- | --- |
| All unintentional injury | V01–X59, Y85–Y86 |
| Transport injuries | V01–V99 |
| Fall | W00–W19 |
| Struck by or against | W20–W22, W50–W52 |
| Cut /pierce | W25–W29, W45 |
| Other specified, classifiable | W23, W35–W41, W44, W49, W85–W91, Y85 |
| Natural or environmental | W42–W43, W53–W64, W92–W99, X20–X39, X51–X57 |
| Drowning | W65–W74 |
| Suffocation | W75–W84 |
| Contact with fire / hot object or substance | X00–X19 |
| Poisoning | X40–X49 |
