# Supplementary material for: Risk factors for unintentional injury hospitalisation among Aboriginal and non-Aboriginal children in Australia’s Northern Territory: A data linkage study
Source: PLoS One. 2024 Nov 5;19(11):e0311586. doi: 10.1371/journal.pone.0311586 (PMC11537399; doi:10.1371/journal.pone.0311586)
Supplement: S2 Appendix — (DOCX) [file pone.0311586.s002.docx]

Appendix Table 2: Comparison of demographic and perinatal characteristics between the study cohort and the excluded cohort, stratified by Aboriginal status

| **Variable** | | **Aboriginal** | | | |  |  |  | **Non-Aboriginal** | | | |  |  |
| --- | --- | --- | --- | --- | --- | --- | --- | --- | --- | --- | --- | --- | --- | --- |
|  |  | **Study cohort** | | **Excluded cohort** | |  | ***p* value** |  | **Study cohort** | | **Excluded cohort** | |  | ***p* value** |
|  |  | n=11442 | 70.4% | n=4819 | 29.6% |  |  |  | n=9747 | 41.7% | n=13605 | 58.3% |  |  |
| **Demography** | |  |  |  |  |  |  |  |  |  |  |  |  |  |
|  | ***Male*** | 5959 | 52.1% | 2473 | 51.3% |  | 0.374 |  | 4939 | 50.7% | 7022 | 51.6% |  | 0.061 |
|  | ***Remoteness*** |  |  |  |  |  | <0.0005 |  |  |  |  |  |  | <0.0005 |
|  | Outer regional | 2453 | 21.4% | 1108 | 23.0% |  |  |  | 7286 | 74.8% | 9427 | 69.3% |  |  |
|  | Remote | 3394 | 29.7% | 1037 | 21.5% |  |  |  | 1863 | 19.1% | 3273 | 24.1% |  |  |
|  | Very remote | 5595 | 48.9% | 2678 | 55.5% |  |  |  | 598 | 6.1% | 910 | 6.7% |  |  |
|  | ***Top End/Central Australia split*** |  |  |  |  |  | 0.001 |  |  |  |  |  |  | <0.0005 |
|  | Top End | 8052 | 70.4% | 3514 | 72.9% |  |  |  | 8451 | 86.7% | 10988 | 80.7% |  |  |
|  | Central Australia | 3390 | 29.6% | 1309 | 27.1% |  |  |  | 1296 | 13.3% | 2622 | 19.3% |  |  |
|  |  |  |  |  |  |  |  |  |  |  |  |  |  |  |
| **Maternal** | |  |  |  |  |  |  |  |  |  |  |  |  |  |
|  | ***Maternal age<20*** | 3040 | 26.6% | 1133 | 23.5% |  | <0.0005 |  | 475 | 4.9% | 399 | 2.9% |  | <0.0005 |
|  | ***Antenatal visits<7*** | 4006 | 35.0% | 1782 | 37.0% |  | 0.019 |  | 1466 | 15.0% | 1790 | 13.2% |  | <0.0005 |
|  | ***Diabetes*** | 1044 | 9.1% | 421 | 8.7% |  | 0.421 |  | 543 | 5.6% | 775 | 5.7% |  | 0.687 |
|  | ***Hypertension*** | 536 | 4.7% | 215 | 4.5% |  | 0.529 |  | 226 | 2.3% | 340 | 2.5% |  | 0.379 |
|  | ***Drank alcohol during pregnancy*** | 1221 | 10.7% | 474 | 9.8% |  | 0.067 |  | 729 | 7.5% | 812 | 6.0% |  | <0.0005 |
|  | ***Smoked during pregnancy*** | 4711 | 41.2% | 1946 | 40.4% |  | 0.620 |  | 1917 | 19.7% | 1932 | 14.2% |  | <0.0005 |
|  | ***Parity*** |  |  |  |  |  |  |  |  |  |  |  |  |  |
|  | <2 | 6494 | 56.8% | 2740 | 56.8% |  | 0.784 |  | 7180 | 73.7% | 10695 | 78.6% |  | <0.0005 |
|  | 2-3 | 3481 | 30.4% | 1443 | 29.9% |  |  |  | 2219 | 22.8% | 2554 | 18.8% |  |  |
|  | 4+ | 1463 | 12.8% | 639 | 13.3% |  |  |  | 343 | 3.5% | 355 | 2.6% |  |  |
|  |  |  |  |  |  |  |  |  |  |  |  |  |  |  |
| **Perinatal** | |  |  |  |  |  |  |  |  |  |  |  |  |  |
|  | ***Twin birth*** | 215 | 1.9% | 83 | 1.7% |  | 0.492 |  | 269 | 2.8% | 367 | 2.7% |  | 0.770 |
|  | ***Pre-term birth*** | 1507 | 13.2% | 775 | 16.1% |  | <0.0005 |  | 682 | 7.0% | 1045 | 7.7% |  | 0.102 |
|  | ***Low birthweight (<2500 grams)*** | 1410 | 12.3% | 1553 | 14.8% |  | <0.0005 |  | 581 | 6.0% | 838 | 6.2% |  | 0.759 |
|  | ***Emergency Caesarean birth*** | 1959 | 17.1% | 774 | 16.1% |  | 0.095 |  | 1383 | 14.2% | 1948 | 14.3% |  | 0.789 |
